# Validation Against Polysomnography of a Transthoracic Impedance Sensor for Screening of Sleep Apnea in Heart Failure Patients: A Pooled Analysis of AIRLESS and UPGRADE

**DOI:** 10.3390/jcm13247519

**Published:** 2024-12-10

**Authors:** Fabian Barbieri, Agne Adukauskaite, Philipp Spitaler, Thomas Senoner, Bernhard Pfeifer, Sabrina Neururer, Peggy Jacon, Sandrine Venier, Sarah Limon, Raoua Ben Messaoud, Jean-Louis Pépin, Florian Hintringer, Wolfgang Dichtl, Pascal Defaye

**Affiliations:** 1 Department of Cardiology, Angiology and Intensive Care, Deutsches Herzzentrum der Charité, 12203 Berlin, Germany; 2 Department of Internal Medicine III, Medical University of Innsbruck, 6020 Innsbruck, Austria; agne.adukauskaite@tirol-kliniken.at (A.A.); philipp.spitaler@i-med.ac.at (P.S.); florian.hintringer@tirol-kliniken.at (F.H.); wolfgang.dichtl@tirol-kliniken.at (W.D.); 3 Helmholtz-Zentrum Hereon, Institute of Active Polymers and Berlin-Brandenburg Center for Regenerative Therapies, 14513 Teltow, Germany; 4 Department of Anaesthesiology and Intensive Care Medicine, Medical University of Innsbruck, 6020 Innsbruck, Austria; thomas.senoner@i-med.ac.at; 5 Institute of Clinical Epidemiology, Tirol Kliniken, 6020 Innsbruck, Austria; bernhard.pfeifer@tirol-kliniken.at (B.P.); s.neururer@tirol-kliniken.at (S.N.); 6 Division for Digital Medicine and Telehealth, University for Health Sciences, Medical Informatics and Technology (UMIT), 6060 Hall in Tirol, Austria; 7 University Hospital of Grenoble Alpes, INSERM U1300, 38043 Grenoble, France; pjacon@chu-grenoble.fr (P.J.); svenier@chu-grenoble.fr (S.V.); slimon@chu-grenoble.fr (S.L.); ext-rbenmessaoud@chu-grenoble.fr (R.B.M.); jpepin@chu-grenoble.fr (J.-L.P.); pdefaye@chu-grenoble.fr (P.D.)

**Keywords:** cardiac implantable electronic devices, sleep apnea, sleep-disordered breathing, screening, polysomnography, implantable cardioverter-defibrillator, cardiac resynchronization therapy, AP scan, ApneaScan, respiratory disturbance index

## Abstract

**Background/Introduction:** Cardiac implantable electronic devices and their integrated thoracic impedance sensors have been used to detect sleep apnea for over a decade now. Despite their usage in daily clinical practice, there are only limited data on their diagnostic accuracy. **Methods:** AIRLESS and UPGRADE were prospective investigator-driven trials meant to validate the AP scan^®^ (Boston Scientific, Marlborough, MA, USA) in heart failure cohorts. Patients, who either fulfilled the criteria for implantation of an implantable cardioverter-defibrillator (ICD), cardiac resynchronization therapy (CRT), or upgrading to CRT according to most recent guidelines at the time of study conduction, were eligible for enrolment. Sleep apnea and its severity, measured by apnea–hypopnea index (AHI), were assessed by polysomnography. For direct comparison, the apnea sensor-derived AP scan^®^ was used from the identical night. **Results:** Overall, 80 patients were analyzed. Median AHI was 21.6 events/h (7.1–34.7), while median AP scan^®^ was 33.0 events/h (26.0–43.0). In the overall cohort, the sensor-derived AP scan^®^ correlated significantly with the AHI (r = 0.61, *p* < 0.001) with a mean difference (MD) of −12.6 (95% confidence interval (CI) −38.2 to 13.0). Furthermore, the AP scan^®^ was found to correlate well with the AHI in patients with obstructive sleep apnea r = 0.73, *p* = 0.011, MD −5.2, 95% CI −22.7 to 12.3), but not central sleep apnea (r = 0.28, *p* = 0.348, MD −10.4, 95% CI −35.4 to 14.6). **Conclusions:** In an exclusive heart failure cohort, the AP scan^®^ correlated well with the PSG-derived AHI. A similar correlation was found in most subgroups except for patients suffering from central sleep apnea.

## 1. Introduction

Cardiac implantable electronic devices (CIED) have become a cornerstone for treatment in modern cardiology. Every year, hundreds of thousands of pacemakers, cardioverter-defibrillators, and devices for cardiac resynchronization therapy (CRT) are implanted for various therapeutical reasons [1,2]. During the last decades, these devices have turned from very basic tools with only minimalistic functions for pacing into extraordinary products with multiple services and diagnostic features. Several of these new features help physicians in their daily clinical lives and are also thought to improve the outcomes of patients. One of them is the AP scan^®^ (Boston Scientific, Marlborough, MA, USA), a rather new technology, which enables continuous monitoring of sleep-disordered breathing (SDB) by measuring differences in thoracic impedance. Such diagnostic tools have become of increasing importance as up to 75% of all patients with severe sleep apnea (SA) are still not diagnosed, whereas nearly 60% of all CIED patients suffer from SA with negative implications on prognosis being mostly observed during long-term observation [3,4,5,6,7].

At this current time, there are several studies describing the usefulness of the AP scan^®^ to detect SDB. Unfortunately, most of the published trials only used respiratory polygraphy for validation, while only a few used polysomnography (PSG), the gold standard for the evaluation of SA. Furthermore, all of these studies included relatively small sample sizes and results should be interpreted cautiously [8,9,10]. Two of the major difficulties, and probably the explanation for small study populations in these investigator-driven trials, are the enormous expanse as well as the high patient burden for the precise assessment of sleep-disordered breathing by performing a PSG. To overcome these issues and to assess diagnostic accuracy among different subgroups, we set out to pool the individual data of two individual trials, AIRLESS and UPGRADE, which have reported the validation of the novel AP scan^®^ by polysomnography in a heart failure patient cohort.

## 2. Materials and Methods

### 2.1. Study Design and Population

This analysis displays pooled data of two prospective investigator-driven trials, AIRLESS (NCT02045173) and UPGRADE (NCT01970423), both aiming to validate the AP scan^®^ in heart failure patients. Each individual trial, as well as the pooled analysis, was conducted in accordance with the requirements of good clinical practice and the ethical principles of the Helsinki Declaration. AIRLESS (Comité de Protection des Personnes, Grenoble, France) and UPGRADE (Ethics Committee of Medical University Innsbruck, Innsbruck, Austria) were approved by their respective local institutional review board/ethics committee, and verbal and written informed consent was obtained from all enrolled patients.

The AP scan^®^ is a software algorithm integrated into CIEDs to monitor and detect SDB. It analyzes fluctuations in thoracic impedance measurements occurring during in- and expiration with the aim of identifying apneas and hypopneas. The exact mechanism is described in previous trials [10]. The methods for polysomnography as well as the inclusion and exclusion criteria of each trial were described in previous publications [6,7,8,9]. In general, both studies focused on heart failure patients receiving an implantable cardioverter-defibrillator (ICD) or CRT device, either with (CRT-D) or without a defibrillator (CRT-P). Patients in AIRLESS solely underwent de novo ICD or CRT-D implantation, while UPGRADE focused on upgrading previously active CIED to CRT-P or CRT-D. Indications for implantation were performed according to the most recent guidelines at the time of study conduction [11].

### 2.2. Endpoint Parameter and Subgroups

The parameter of choice to validate the AP scan^®^ was the PSG-derived apnea–hypopnea index (AHI). In contrast to previously presented publications of the UPGRADE trial and to homogenize their definitions, an AHI of 15 or more was used as a cut-off for the presence of SA. SA was defined as central sleep apnea (CSA) syndrome whenever the total number of central apneas or hypopneas exceeded 50% of the total number of apneas and hypopneas. Similarly, SA was described as obstructive sleep apnea (OSA) syndrome whenever apneas or hypopneas were predominantly of obstructive type. The presence of chronic obstructive pulmonary disease (COPD) was determined according to the GOLD criteria or the permanent use of inhaled/oral bronchodilatory or steroid therapy aimed at lung disease. Overweight was defined as a body mass index of 25 kg/m^2^ and above.

### 2.3. Statistics

Continuous variables are expressed as a median and interquartile range, and categorical variables are reported as a number and percentage. Differences in categorical variables will be compared by using the chi-square test. Distribution will be assessed by the Shapiro–Wilk test and by reviewing corresponding histograms. The coefficient for intermodality correlations (Spearman and Pearson) was chosen depending on the underlying distribution. Divergences in diagnostic accuracy between continuous variables were assessed by using the Bland–Altman method, whereas a linear regression was used to analyze the slope of the regression line and consequently evaluate any form of proportionality bias [12]. Graphics were designed by using GraphPad PRISM, version 5 (GraphPad Software, Inc., La Jolla, CA, USA), whereas statistical analysis was conducted with IBM SPSS, version 21 (IBM Corporation, Armonk, NY, USA). *p*-values ≤ 0.05 were considered statistically significant.

## 3. Results

### 3.1. Study Population and Baseline Characteristics

Overall, 80 patients were included in this analysis. The majority were male (*n* = 67, 83.8%) with a median age of 73 years (interquartile range: 65–77). Median body mass index was found to be 26.2 (23.7–28.4), while 47 (58.8%) patients were obese. A total of 16 (20.0%) patients received a conventional ICD system, 21 (26.3%) were implanted with a CRT-P, and 43 (53.8%) with a CRT-D. A total of 54 patients (67.5%) received an upgrade to a CRT system, and 26 (32.5%) were de-novo implantations. Exactly half of the patients (*n* = 40, 50.0%) were known to suffer from atrial fibrillation, while only a minority (*n* = 16, 20.0%) were known to have COPD. Diagnosis of CSA was established in 29 (36.3%) patients, and OSA was yielded in 17 (21.3%) patients. Periodic leg movement syndrome (PLMS) was present in 45 (56.3%) patients. Median AHI was 21.6 events/h (7.1–34.7), while median AP scan^®^ was 33.0 events/h (26.0–43.0). Creating an average of all available measurements for 15 days (including 7 nights prior/post index night) or 31 days (including 15 nights prior/post index night) resulted in a median AP scan^®^ of 31.5 events/h (24.5–41.4) and 31.8 events/h (24.2–41.9).

### 3.2. Correlation Between AHI and AP Scan^®^

**Figure 1:**
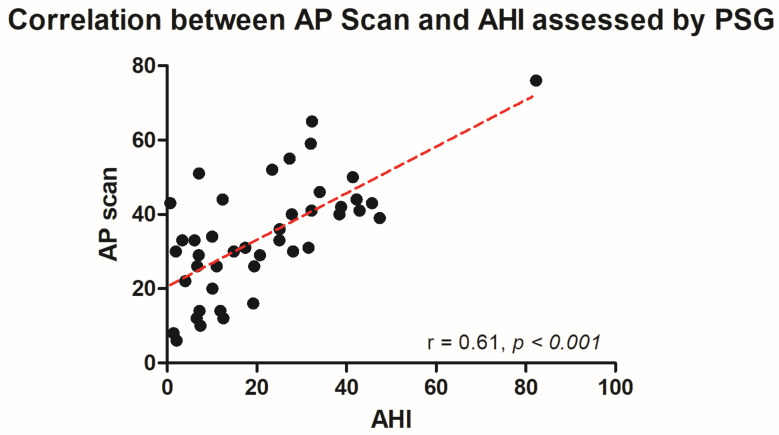
Scatter plot showing the correlation between the device-derived AP scan^®^ and the apnea–hypopnea index.

**Figure 2:**
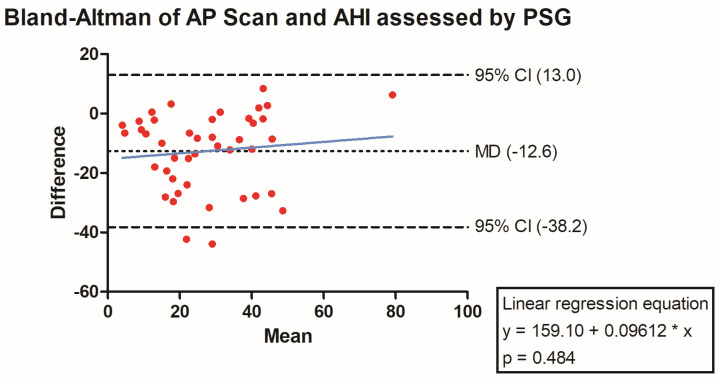
Bland–Altman plot showing the overestimation of the device-derived AP scan^®^ compared to the apnea–hypopnea index by visualizing the mean of differences with their respective 95% confidence interval. *Y*-axis depicts the difference between the polysomnography-derived apnea–hypopnea index and the AP scan^®^, while the *x*-axis plots their mean. The fitted linear regression line demonstrates no form of proportionality bias.

**Figure 3:**
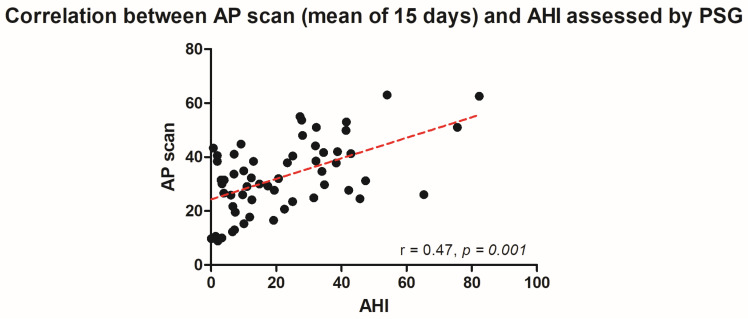
Scatter plot showing the correlation between the mean of 15 device-derived AP scan^®^ measurements and the apnea–hypopnea index.

**Figure 4:**
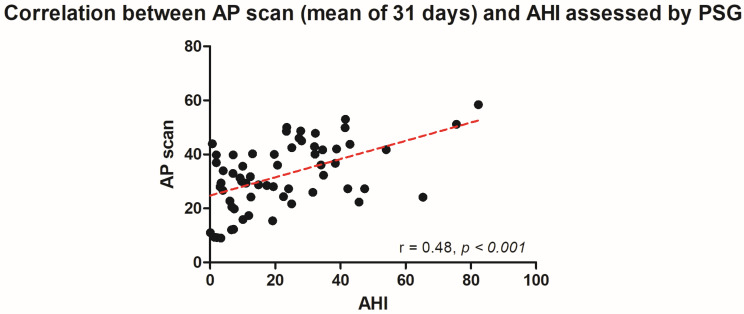
Scatter plot showing the correlation between the mean of 31 device-derived AP scan^®^ measurements and the apnea–hypopnea index.

**Figure 5:**
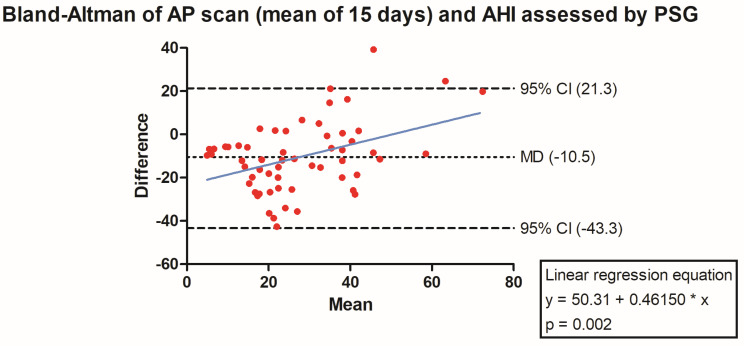
Bland–Altman plot showing the overestimation of the mean of 15 device-derived AP scan^®^ measurements compared the apnea–hypopnea index by visualizing the mean of differences with their respective 95% confidence interval. *Y*-axis depicts the difference between the polysomnography-derived apnea–hypopnea index and the mean of 15 AP scan^®^ measurements, while the *x*-axis plots their mean. The fitted linear regression line demonstrates a higher form of overestimation with smaller apnea–hypopnea index measurements.

**Figure 6:**
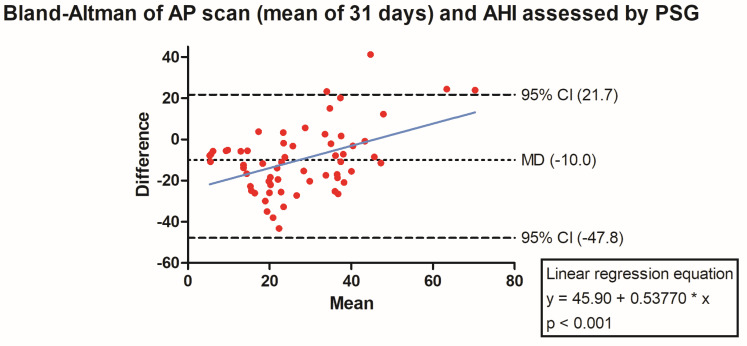
Bland–Altman plot showing the overestimation of the mean of 31 device-derived AP Scan^®^ measurements compared the apnea–hypopnea index by visualizing the mean of differences with their respective 95% confidence interval. *Y*-axis depicts the difference between the polysomnography-derived apnea–hypopnea index and the mean of 31 AP scan^®^ measurements, while the *x*-axis plots their mean. The fitted linear regression line demonstrates a higher form of overestimation with smaller apnea–hypopnea index measurements.

The PSG-derived AHI and the AP scan^®^ measurement during the exact same night correlated very well in the overall cohort (r = 0.61, *p* < 0.001, Figure 1) with moderate overestimation of the true value by the AP scan^®^ (mean difference (MD) of −12.6, 95% confidence interval (CI) −38.2 to 13.0, Figure 2). There was no proportionality bias found during the assessment of the fitted linear regression slope (y = 159.10 + 0.09612 × x, *p* = 0.484). To take multiple measurements into account, an average of all available measurements was created for 15 days and 31 days. Neither concept was able to improve the diagnostic accuracy as both correlated with the AHI in a similar way (r = 0.47, *p* < 0.001 and r = 0.48, *p* < 0.001, Figure 3 and Figure 4) with nearly identical overestimation by the AP scan^®^ (MD of −10.5, 95% CI −43.3 to 21.3 and MD of −10.0, 95% CI −47.8 to 21.7, Figure 5 and Figure 6). Conversely, to the single-night measurements, a proportionality bias was found when assessing the linear regression slopes (y = 50.31 + 0.46150 × x, *p* = 0.002 and y = 45.90 + 0.53770 × x, *p* < 0.001), indicating a higher form of overestimation with lower AHI measurements.

### 3.3. Subgroup Analysis

Multiple subgroups were assessed regarding their correlation between the AHI and AP scan^®^. The AP scan^®^ performed very well in females (r = 0.71, *p* = 0.047, MD of −6.1, 95% CI −26.4 to 14.2). A significant correlation was also found for male patients (r = 0.52, *p* = 0.001), yet with a slightly higher overestimation by the AP scan^®^ (MD of −14.1, 95% CI −40.1 to 11.9). Hardly any differences in correlation were found regarding the implanted type of device (ICD recipients: r = 0.67, *p* < 0.001, MD of −11.2, 95% CI −36.6 to 14.2 versus CRT recipients: r = 0.62, *p* < 0.001, MD of −13.5, 95% CI −40.2 to 13.2) and indication for implantation (de-novo implantations: r = 0.64, *p* = 0.003, MD of −11.3, 95% CI −33.4 to 10.9 versus upgrading to CRT: r = 0.59, *p* = 0.003, MD of −13.7, 95% CI −42.0 to 14.6). Furthermore, the AP scan^®^ performed particularly well in overweight patients (r = 0.75, *p* < 0.001, MD of −7.5, 95% CI −29.5 to 14.5), whereas it failed to correlate with the AHI in probands suffering from COPD (r = 0.20, *p* = 0.747, MD of −16.9, 95% CI −38.1 to 4.3). The presence of a PLMS did not affect the diagnostic accuracy of the AP scan^®^ (r = 0.60, *p* = 0.007, MD of −16.1, 95% CI −43.9 to 11.8). Analysis of both SA subtypes showed very divergent results. Whereas a strong correlation between the AHI and AP scan^®^ was observed in OSA patients (r = 0.73, *p* = 0.011, MD of −5.2, 95% CI −22.7 to 12.3), no correlation was detected in probands suffering from CSA (r = 0.28, *p* = 0.348, MD of −10.4, 95% CI −35.4 to 14.6).

## 4. Discussion

By combining the datasets of two previously reported studies, the presented analysis aimed at further validating the AP scan^®^ and assessing diagnostic accuracy in multiple subgroups. The most important findings were as follows:

(1) There was a good correlation between the PSG-derived AHI and the AP scan^®^ in the overall cohort with a moderate overestimation by the AP scan^®^.
(2) Adequate diagnostic accuracy was observed throughout many subgroups, particularly in females and overweight patients.
(3) It is the first study to differentiate the diagnostic accuracy in subtypes of SA showing a strong correlation between the AHI and AP scan^®^ in OSA, but a rather poor one in CSA.
(4) No correlation was found in patients suffering from COPD suggesting that measurements in such patients are prone to error.
(5) Taking multiple measurements into account did not improve the diagnostic accuracy and showed furthermore a higher form of overestimation with smaller AHI values during the index night.

Many patients with SA remain undiagnosed due to the hurdles involved in screening. In particular, the high expense involved in carrying out polysomnographies paired with the high incidence of SDBs leads to an undersupply within the general population and thus demonstrates the need for alternative methods of screening [13]. This problem has also reached research facilities as there are even studies using questionnaires like the Epworth Sleepiness Scale to define the presence of SDB [14]. Unfortunately, such self-reported questionnaires have limited predictive value and are prone to miss SA [15]. CIED has emerged as a potential alternative and entails the opportunity to partially solve this issue with their respective algorithms. Currently, there are two algorithms available. The SAM^®^ algorithm by MicroPort CRM (Shanghai, China; formerly Sorin) was introduced first after which the AP scan^®^ was brought to the market by Boston Scientific. SAM^®^ was initially validated by the DREAM study, while the AP scan^®^ was investigated by three separate investigator-driven trials [8,9,10,16]. In general, though, all presented studies are fairly small, with a maximum of 41 patients included, and thus leave many questions unanswered. To the best of our knowledge, the presented analysis is currently the largest reported study assessing the diagnostic accuracy of the AP scan^®^ by using PSG, the gold standard for evaluation of SDBs. The yielded correlation between the AHI and AP scan^®^ was similar to previously reported ones by Chen et al. and the AIRLESS study, while, in addition, the amount of overestimation in events per hour is comparable to the SAM^®^ algorithm (9.2 events/h in DREAM versus 12.6 events/h in presented analysis for AP scan^®^) [8,10,16]. With the increased patient number, however, subgroup analysis was possible for the first time. Here, the AP scan^®^ was able to demonstrate adequate diagnostic accuracy in most subgroups. There was no difference regarding sex with an even stronger correlation coefficient in female patients. Interestingly, a distinct difference between OSA and CSA was observable. While patients suffering from OSA yielded a strong correlation (r = 0.73) between the AHI and AP scan^®^, there was hardly any correlation in CSA patients (r = 0.28). This might be explained by their pathophysiology resulting in different breathing patterns. CSA is typically characterized by an absent drive to breathe, whereas continuous respiratory efforts are found in OSA [17]. The strong correlation between the AHI and AP scan^®^ in OSA might also explain the predictive value for new-onset and prolonged episodes of atrial fibrillation as the interplay between OSA and atrial fibrillation is already well-known [18]. Another subgroup with suboptimal diagnostic accuracy was patients with known COPD. This might be due to the overlap with other co-morbidities like pulmonary hypertension and heart failure, each often associated with Cheyne–Stokes respiration and potentially being a relevant confounder [19,20,21]. The lack of exclusion of COPD patients and the predominance of patients suffering from CSA are, therefore, also one of the main reasons why the UPGRADE trial yielded a worse correlation than other studies that investigated the AP scan^®^. The type of implanted device, however, did not affect the diagnostic accuracy, which stands in analogy to previously reported results [22].

Interestingly, the diagnostic value of the AP scan^®^ was also assessed in other trials by utilizing different methodologies. Home sleep monitoring by polygraphy, for example, was found to correlate very well (r = 0.80, *p* < 0.001) with the AP scan^®^, demonstrating a similar form of overestimation by the CIED algorithm (11 events/h) [23]. Although polygraphy represents the closest tool to a real-life scenario, it is still thought that it may underestimate the severity of sleep apnea by potentially yielding lower AHI values compared to an in-laboratory test due to its inability to uncover arousal-related apneas and hypopneas [24]. A same-night comparison of the AP scan^®^ and polygraphy-derived AHI was also assessed as a secondary endpoint in the DASAP-HF study. In the 49 patients analyzed, a similar correlation was found (r = 0.74, *p* < 0.001), again demonstrating alike bias between both methodologies with an overestimation of 11 events/h by the AP scan^®^ [25]. Beyond its accuracy measured by PSG and polygraphy, the AP scan^®^ was also found to be predictive of heart failure development in a small, retrospective trial [26]. Larger trials ought to confirm this and assess its predictive value on malignant ventricular tachyarrhythmias, stroke, heart failure hospitalizations, mortality, and quality of life are currently still ongoing [27]. A recent meta-analysis has proposed the predictive effect of SDB on ventricular tachyarrhythmias and consequent ICD therapies in heart failure patients, although the methodology for establishing diagnosis in included trials was non-homogenous and included both PSG and polygraphy [28]. It remains to be seen whether the AP scan^®^ has similar predictive value and could also help in further defining patients who might be candidates for ICD implantation as currently used markers and tools seem to be insufficient in both ischemic and non-ischemic cardiomyopathy [29,30]. Night-time variability in SA severity could also be of importance in this setting, as already shown by previous trials assessing the predictive value of atrial fibrillation occurrence and duration [31,32,33].

To assess whether single-night fluctuations of the AP scan^®^, a common finding in clinical routine, may be ameliorated by taking an average of multiple measurements, a mean was formed out of 15 and 31 days, each surrounding the index night. Already by inspecting the minor difference between median values of single (33.0 events/h) and formed average of repetitive (31.5 events/h and 31.8 events/h)-night measurements, it became evident that the single-night measurement is reflective of repetitive measurements. There was hardly any difference during the comparison of the mean difference and consequently the amount of overestimation in the Bland–Altman analysis. Nonetheless, a proportionality bias was uncovered showing that the degree of overestimation by taking multiple AP scan^®^ measurements into account was more pronounced with lower AHI values.

## 5. Conclusions

In conclusion, the AP scan^®^ did correlate well with the PSG-derived AHI in an exclusive heart failure cohort. A similar correlation is found in most subgroups with moderate overestimation of the true value; the most important exceptions seem to be patients suffering from CSA and COPD. Besides its value for screening of SDB, the AP scan^®^ may also be used for long-term monitoring of patients with OSA.

## 6. Limitations

Although the presented analysis reflects one of the largest cohorts investigating the accuracy of the AP scan^®^ by the means of PSG so far, the overall number of participants remains small, and the possibility of inherent bias cannot be excluded. Similar to other trials involving PSG, the expense of this diagnostic tool poses a significant challenge in conducting larger prospective trials. Another limiting factor is the difference in inclusion and exclusion criteria between both trials. In UPGRADE, only patients with a previously implanted CIED system were allowed, whereas AIRLESS enrolled de novo implantations of either an ICD or CRT-D system. Lastly, this analysis included only heart failure patients. It remains to be shown whether the diagnostic accuracy of the presented subgroups is different in other patient populations.

## Data Availability

The data underlying this article will be shared on reasonable request to the corresponding author.
